# P-1788. Impact of standardized audit and feedback script and guide to reduce empiric fluoroquinolone use at two community hospitals

**DOI:** 10.1093/ofid/ofae631.1951

**Published:** 2025-01-29

**Authors:** Evan Hurley, Lauren Warren

**Affiliations:** Inova Health System, Washington , District of Columbia; Inova Health System, Washington , District of Columbia

## Abstract

**Background:**

Fluoroquinolones are one of a few oral options for drug-resistant gram-negative infections. Preservation of their susceptibility is an important goal for many antimicrobial stewardship programs. There are a multitude of antimicrobial stewardship efforts to reduce their use in hospitalized patients with community-acquired infections to reduce resistance and prevent adverse effects. At our institutions, fluoroquinolone susceptibility rates are lower than guideline recommendations for use in many community-acquired infections warranting targeted interventions in empiric use.

**Figure:**
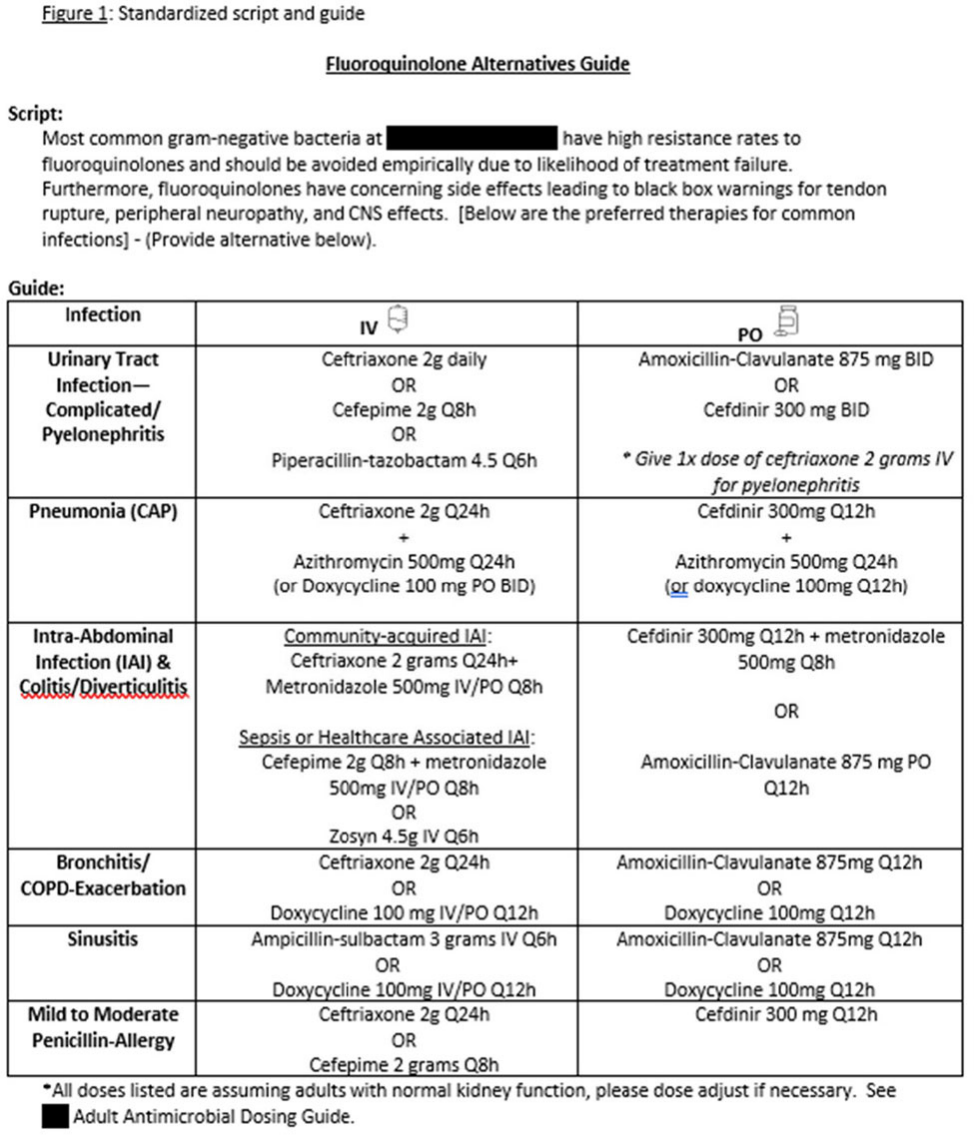

Standardized script and guide used by pharmacists during daily audit and feedback of fluoroquinolones in hospitalized adult patients.

**Methods:**

At two community hospitals in northern Virginia, a standardized script and guide were created and implemented in May of 2023 for use in prospective audit and feedback of fluoroquinolones in hospitalized adult patients (Figure 1). The script and guide are used by pharmacists during daily antimicrobial stewardship monitoring and order verification to reduce the use of fluoroquinolones in the empiric treatment of community-acquired infections. Interventions were made by pharmacists either in person or via electronic communication in the electronic health record (EHR). Interventions were documented in the EHR for future analysis. A pre-implementation period of May 2022 to April 2023 was compared to a post-standardized script implementation period of May 2023 to April 2024.

**Figure 2:**
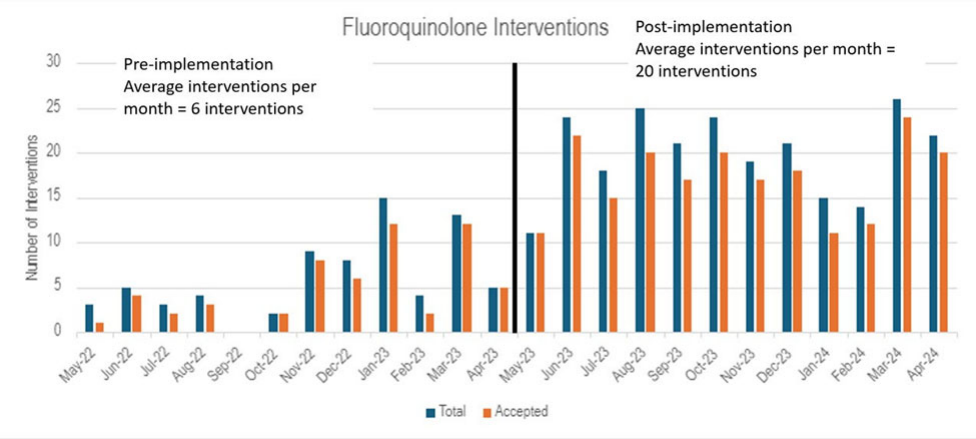
Fluoroquinolone interventions

Fluoroquinolone interventions by month from May 2022 through April 2024. Interventions were documented by pharmacists during daily antimicrobial stewardship monitoring in hospitalized adult patients. Pre-implementation, the average number of interventions per month was 6. Post-implementation, the average number of interventions per month was 20.

**Results:**

A total of 71 documented interventions were made between May 2022 to April 2023 prior to implementation of the standardized script and guide. After the standardized script and guide were implemented, a total of 240 interventions were documented between May 2023 and April 2024 (Figure 2). After implementation, there was an average monthly increase of 16 interventions per month. The acceptance rate of interventions was 80% for pre-implementation and 86% post-implementation. Monthly average day of therapy per 1000 patient days present (DOT/1000) decreased post-implementation from 41 (DOT/1000) to 31 (DOT/1000), a difference of 10 DOT/1000 (p< 0.05) (Figure 3).

**Figure 3:**
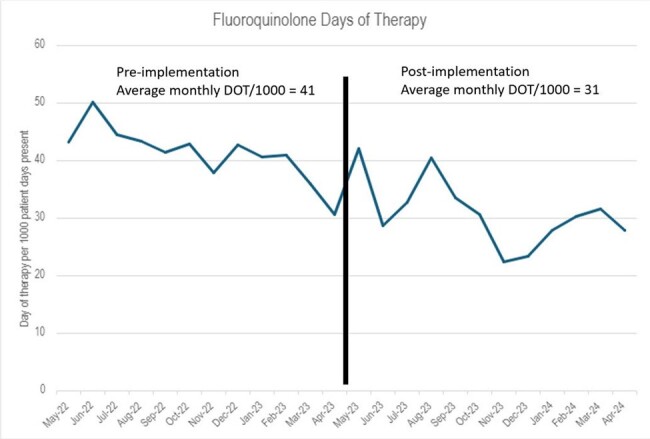
- Days of therapy per 1000 patient days present for fluoroquinolones

The days of therapy per 1000 patient days (DOT/1000) for fluoroquinolones from May 2022 through April 2024. Pre-implementation the average monthly DOT/1000 was 41 and post-implementation the average monthly DOT/1000 was 31 a decrease of 10 DOT/1000 (p<0.05).

**Conclusion:**

A standardized script and guide can reduce inpatient fluoroquinolone use for community-acquired infections in hospitalized adults when used by pharmacists during daily antimicrobial stewardship monitoring.

**Disclosures:**

**All Authors**: No reported disclosures

